# Pre-Processing of Panchromatic Images to Improve Object Detection in Pansharpened Images

**DOI:** 10.3390/s19235146

**Published:** 2019-11-24

**Authors:** Aleksandra Sekrecka, Michal Kedzierski, Damian Wierzbicki

**Affiliations:** Department of Remote Sensing, Photogrammetry and Imagery Intelligence, Institute of Geodesy, Faculty of Civil Engineering and Geodesy, Military University of Technology, 00-908 Warsaw, Poland; michal.kedzierski@wat.edu.pl (M.K.); damian.wierzbicki@wat.edu.pl (D.W.)

**Keywords:** data fusion, pansharpening, features detector, object detection, spectral quality

## Abstract

In recent years, many techniques of fusion of multi-sensors satellite images have been developed. This article focuses on examining and improvement the usability of pansharpened images for object detection, especially when fusing data with a high GSD ratio. A methodology to improve an interpretative ability of pansharpening results is based on pre-processing of the panchromatic image using Logarithmic-Laplace filtration. The proposed approach was used to examine several different pansharpening methods and data sets with different spatial resolution ratios, i.e., from 1:4 to 1:60. The obtained results showed that the proposed approach significantly improves an object detection of fused images, especially for imagery data with a high-resolution ratio. The interpretative ability was assessed using qualitative method (based on image segmentation) and quantitative method (using an indicator based on the Speeded Up Robust Features (SURF) detector). In the case of combining data acquired with the same sensor the interpretative potential had improved by a dozen or so per cent. However, for data with a high resolution ratio, the improvement was several dozen, or even several hundred per cents, in the case of images blurred after pansharpening by the classic method (with original panchromatic image). Image segmentation showed that it is possible to recognize narrow objects that were originally blurred and difficult to identify. In addition, for panchromatic images acquired by WorldView-2, the proposed approach improved not only object detection but also the spectral quality of the fused image.

## 1. Introduction

The development of image acquisition techniques expands the possibilities of their application. Imagery obtained from various altitudes (i.e., satellite, aerial or unmanned aerial vehicles) are used for multiple analysis in many fields of science and technology, such as urban planning and the environmental monitoring [1,2], archeology [3], land-use and landcover mapping [4,5].

Each image is described by its resolution; the most important in remote sensing are spectral and spatial resolution. The spectral resolution depends on the number and width of the spectral ranges in which the image is acquired. The spatial resolution is defined by the distances of the neighbouring pixels–Ground Sampling Distance (GSD) [6]. Availability of a variety of imagery data affects attempts of their integration. Pansharpening—the process of combining a high-resolution panchromatic image (*PAN*) with a low-resolution multispectral image (MS)—allows one to obtain images with both high spatial and spectral resolution. Most often, the integration of the images is performed for data acquired by sensors mounted on the same platform for which the GSD ratio ranges from 1:2 to 1:5 [7] (from now on this ratio range is referred as the standard one). There are many approaches to the integration of such data, from which two main groups can be distinguished: component substitution and multiresolution. The most common methods based on the replacement of components are Principal Component Analysis (PCA) [8], Gram-Schmidt (GS) [9,10], and Intensity-Hue-Saturation (IHS) [11]. Principal Component Analysis and Gram-Schmidt orthogonalisation can work on images with any number of bands, while IHS transformation is limited to only three image bands. Therefore, new approaches have been developed to extend this method for any number of spectral channels. One of the solutions is Generalized IHS (GIHS) [12] and a similar solution taking into account Brovey colour standardisation (GIHS-BT) [12,13]. The multiresolution group includes the method based on high-pass filtration (HPF) [14], wavelet transformation [15,16], generalized Laplacian pyramids with modulation transfer function matched filtering (MTF-GLP) [17,18,19] or algorithms based on modulation transfer function and spatial principal component analysis (SPCA) [20]. Recently, new approaches to classical methods and hybrid methods have been developed, such as Partial Replacement Adaptive Component Substitution PRACS [21], Bayesian data fusion [22,23], Structure Tensor-Based Algorithm [24], pansharpening using guided filtering (GF) [25]. Moreover, there is a new group of methods based on neural networks and deep learning, which are represented by approaches such as PanNet [26], pan-sharpening method based on a convolutional neural network CNN [27], deep self-learning (DSL) network for adaptive image pansharpening [28]. Each method gives different results, which are considered mainly in terms of spectral and spatial quality. The state-of-the-art pan-sharpening methods give both high spectral and spatial quality. We focus on exploring the usability of various pansharpened images for object detection, especially by automated algorithms that are based on detectors of characteristic points in the image. Then, in addition to high spectral and spatial quality, proper image radiometry is also needed. As a result of pansharepening (mainly when combining data with a high GSD ratio), there are often blurred and locally low contrast areas. In this manuscript, we propose a methodology of panchromatic image pre-processing that allows it to be sharpened with simultaneous local changes in radiometry and used to improve the interpretation capabilities of the image after fusion.

In recent years, there have been attempts to integrate data with a very high GSD ratio obtained from various sensors [29,30,31,32,33]. Classic solutions in such cases often produce low-quality results, which is why it is essential to develop an approach that will improve this quality. In [30,32], a two-stage integration has been proposed, where in the first stage the panchromatic image is modified, and then the fusion is performed. The first approach [30] proposes modifications of the panchromatic image using a weighting factor with a constant value of 0.1. The second one selects the pixels representing spatial elements and calculates weights based on the distance of each pixel from the nearest spatial element [32]. Both approaches aim to increase the spectral quality of integration of images with a high spatial resolution ratio. Our approach is inspired by the mentioned research but has a different purpose. In this manuscript we propose a methodology for pre-processing a panchromatic image that aims at improving the interpretative ability of not only data fusion with a standard GSD ratio but above all, it is dedicated to non-standard data.

Achieving high ability to objects identification requires an appropriate method of spatial objects selection. The most popular way to extract details in the image is high-pass filtration. The simplest filters are gradients that are based on the first derivative, and operate directionally. They are suitable for detecting linear elements. For detection of non-linear objects, non-directional Laplace filters based on the second derivative are recommended. Both are the basis for many methods of extracting details in the image. One of the most common filters for edge detection is the Canny filter [34] that applies the Sobel convolution in the vertical and horizontal direction after the image denoising with the Gaussian filter with a kernel of size 5 × 5. Next, the directions are calculated and aligned to the angles of 0°, 45°, 90° and 135°, after which non-maximum suppression and hysteresis thresholding lead to the final detection of the edges. Canny’s filter, as well as most of the filters, gives good results when detecting clearly visible edges. The edge in the image is defined as a sudden change in the signal frequency, and therefore, the higher the contrast around the edge, the easier it is to identify. In the satellite images often have narrow edges (1–2 pixels) and places where the contrast between the background and the narrow edge is too low. Enhancing such edges is essential for achieving the high interpretative -ability of pansharpening, especially in the case of data with a high GSD ratio. The approach described in this manuscript involves the development of a filtration method that will allow enhancing spatial details, in particular, linear objects with a width of 1–2 pixels.

## 2. Materials 

The tests were carried out for three data sets with different GSD ratios. The first set is panchromatic and multispectral images acquired by WorldView-2 (delivered by DigitalGlobe in Colorado, USA) -a Very High-Resolution Satellite (VHRS) that acquires panchromatic data with a resolution of 0.5 m and multispectral data with a resolution of 2 m [35]. The GSD ratio is therefore 1:4 and this is a commonly used data set for the pansharpening purpose. The second dataset also contains a panchromatic image from WorldView-2, but this time integrated with the low-resolution multi-spectral image from the Landsat 7 ETM satellite (available from USGS) with GSD equal to 30 m [36]. The ratio of spatial resolution is, therefore very high, and it is 1:60. The next dataset also has a high GSD ratio (1:30). Multispectral image from the Landsat 8 OLI satellite (available from USGS, GSD = 30 m) [36] is fused with a panchromatic image from the satellite IKONOS-2 (delivered by DigitalGlobe in Colorado, USA, GSD = 1 m). The data from set 1 were obtained at the same moment, under the same lighting conditions. However, the data from sets 2 and 3 were selected in such a way that they would be acquired in the nearest possible lighting and weather conditions. However, it is usually impossible to obtain scenes from the Landsat satellite recorded entirely at the same time as the scenes from WorldView-2 or IKONOS-2. The integration of such data is a big challenge due to the need for a combination of images acquired by various sensors, usually in different conditions and with a high-resolution difference. 

**Figure 1:**
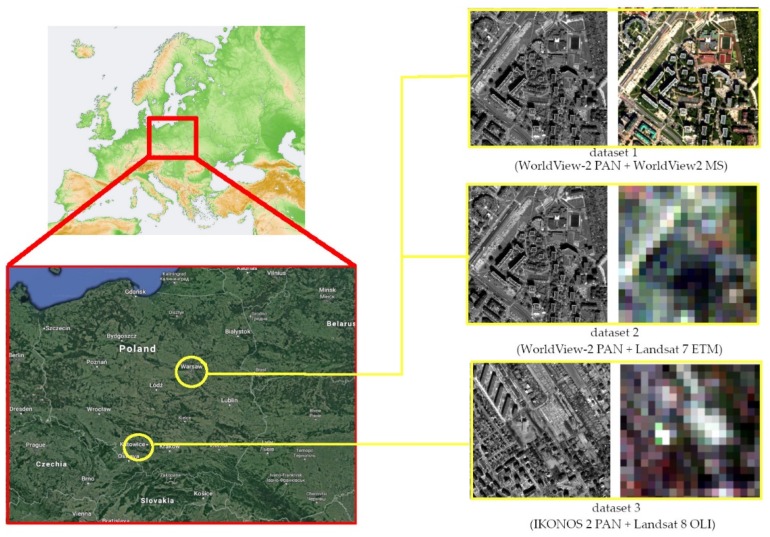
Data sets and their location. Set 1: the panchromatic and the multispectral image from the WorldView-2 satellite; Set 2: panchromatic image from the WorldView-2 and multispectral satellite from the Landsat 7 ETM satellite; Set 3: panchromatic image from the satellite IKONOS-2 and multispectral from the satellite Landsat 8 OLI.

This often results in spectral distortions and/or blurred image after the fusion. The panchromatic image acquired by IKONOS satellite has a twice lower spatial resolution than the panchromatic image acquired by the WorldView-2 satellite, so the spatial details are mapped less precisely in it. The choice of such data sets allowed the verification of the proposed approach for various GSD ratios (from standard to very high) and different spatial resolution of a high-resolution image. All data show areas in Poland. Sets 1 and 2 represent a lowland area, with a highly urbanised area of the capital of Poland (Warsaw). Whereas, set 3 shows the upland area, which is medium urbanised and partly forested. All testing images are 1200 by 1200 pixels. Data sets are shown in Figure 1.

The selection of data from different regions made it possible to verify the correctness of the proposed approach for images with different land cover. The greater the diversity of pixel values in the neighbourhood, the greater the risk of spatial and spectral distortions.

## 3. Methods 

This manuscript proposes a solution that allows sharpening of even narrow edges in areas of low contrast. Since the aim was to expose narrow lines and small details, there was a risk that the noise (whose size is usually 1 or 2 pixels) can be treated as a small detail. For this reason, the image should be denoised first. Such an approach is known in image processing and used for example, in the Canny filter [34]. The noise reduction stage is essential and necessary because noise is always present in every image. It is not always visible to the naked eye, but it is always advisable to reduce it before extracting details. Canny’s filter, as well as many other solutions, propose the use of the Gauss function, but this function not only reduces noise but also blur the edges, which may further complicate their detection, especially if they are lines with a width of 1–2 pixels. It is assumed in Gauss filters that images have smooth spatial variations, and pixels in the neighbourhood have very similar values because averaging the pixel values in the local area suppresses noise while maintaining the features in the image. However, this assumption fails for the edges, where the spatial variations are not smooth, and the use of a Gauss filter causes the edges to blur. This problem is solved by the Bilateral Filter by filtering in two areas of the spectral domain. That is local, nonlinear and noniterative technique, which takes into account both the similarity of grey levels and the geometric proximity of neighbouring pixels [37,38]. The GBMTF method is a combination of Gaussian filtration and Bilateral Filtration, where, after the wavelet decomposition, frequency thresholding and wavelet reconstruction takes place [37]. Therefore, the proposed approach uses the GBMFT noise reduction method (Gaussian Bilateral Filter and its Method Noise Thresholding).

**Figure 2:**
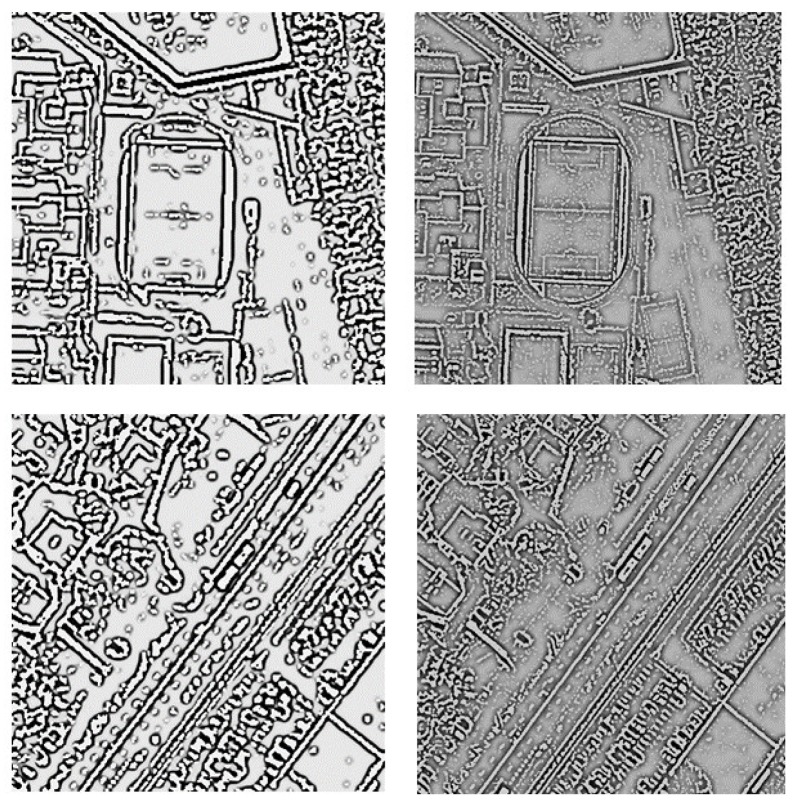
Examples of filtration results before resampling (on **left)** and after 16 times resampling (on **right**).

To preserve the linearity of the narrow edges during filtration, it is recommended that before the high-pass filtration, the integrated images should be resampled to 16-times smaller pixel size. This approach will not introduce new information, but some edges will be now represented not by 1–2 pixels, but by 16–32, which significantly improves their detection (Figure 2).

Moreover, the creation of 16 pixels from one allows maintaining the continuity of newly-detected edges. With 4-fold resampling, some edges were incomplete and broken so that they could be lost during further processing of the image. High-pass filtration using the Laplace filter integrated with the logarithmic function was used to detect the edges (Logarithmic-Laplace filtration-LL). The application of logarithm allows local changes of the histogram in the vicinity of narrow invisible edges, which further enables their detection. The image is processed according to the following equation:

(1) $$PAN_{F}{}_{\left(x,y\right)}=\frac{\sum_{i}\sum_{j}f_{\left(i,j\right)}(\log\left(PAN\prime_{\left(x+i-1,y+j-1\right)}\right))}{N}$$

where $PAN_{F}$ is a panchromatic image after filtration, f means convolution function, PAN′ is a panchromatic image after noise reduction and up-sumpling, x,y are pixel coordinates in the image, (i,j) are the coordinates of the kernel elements and the central element has coordinates (1, 1). N is the number of all pixels in the kernel area (so N is always larger than 0).

The level of image sampling determines the size of the kernel. As in the study of the spatial quality of sharpened images, it is recommended that the size of the kernel be 2*r* + 1, where *r* is the ratio of the pixel size of the images before and after resampling [39]. Thus, with a 16-fold resampling, the kernel size should be 33. In the $PAN_{F}$ image, thresholding was performed to extract only the edges that would later be used to modify the panchromatic image. The threshold value was determined based on the quartiles describing the distribution of pixel values. The median is the second quartile $\left(Q_{2}\right)$ and it determines the value below and above which 50% of the data is located:

(2) $$Q_{2}=Median=\left\{\begin{array}{ll} x_{\frac{n+1}{2}}, & where\;n\;is\;odd\\ \frac{1}{2}\left(x_{\frac{n}{2}}+x_{\frac{2}{2}+1}\right), & where\;n\;is\;even \end{array}\right\}$$

where n is the number of elements in the test sample. The threshold range defining the edges was the third quartile ($Q_{3}$), calculated according to the Formula (2) as a median from $Q_{2}$ to the maximum value. The third quartile allows selecting only those pixels whose values were higher than 75% of the others. That enabled the detection of the actual edges of objects and the elimination of less important edges often resulting from changes in intensity caused by image sampling. The image has been closed (the erosion of dilatation) [40] to remove noise in the mask with edges (image after LL filtration is denoted as $PAN_{F}$ and image after closing is denoted as $PAN_{F}{}_{cl\left(x,y\right)}$.

The new panchromatic image PAN″ image preserves the original values for non-edge pixels (i.e., values after filtration lower than the original and/or lower than the third quartile) and modified values for pixels belonging to the edges. The new values for pixels representing the edges are the arithmetic mean of the original and post-processed values according to the Formula (1) so that the edges are brightened and clearly visible in the image. Finally, the modified panchromatic PAN″ picture takes the form according to Equation (3)

(3) $$PAN{\prime \prime }_{\left(x,y\right)}=\left\{\begin{array}{ll} PAN_{\left(x,y\right)}, & PAN_{Fcl(x,y)}<Q_{3}\;or\;PAN_{Fcl(x,y)}<PAN_{\left(x,y\right)}\\ 0.5\cdot (PAN^{}{}_{\left(x,y\right)}+\log(PAN^{\prime }{}_{\left(x,y\right)})), & PAN_{Fcl(x,y)}\geq Q_{3}\;and\;PAN_{Fcl(x,y)}\geq PAN_{\left(x,y\right)} \end{array}\right\}$$

where PAN is the pixel values in the original panchromatic image (after up-sampling), $PAN_{F}{}_{cl\left(x,y\right)}$ panchromatic image after LL filtration and image closing and (x,y) determines the pixel coordinates in the image.

The development of a mask for detecting edges was necessary because modifying the whole image would result in changes in spectral properties and a significant deterioration in the quality of spectral integration. However, modifying only the edges will not significantly affect the spectral quality of the image, because the modified pixels constitute no more than 20% of all pixels in the image. The research was carried out for a fragment of the highly urbanized area scene, so it can be expected that for other data the percentage of modified pixels will be similar to the given or smaller (e.g., for flat and undeveloped areas).

**Figure 3:**
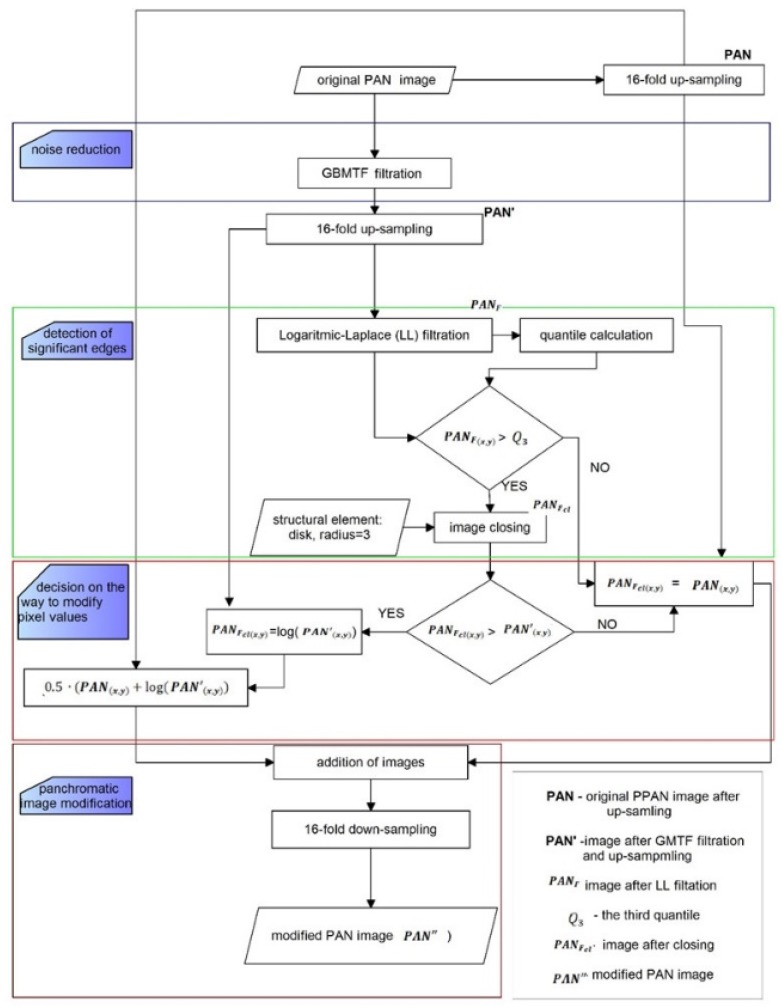
Flowchart of proposed approach of panchromatic image modifiaction.

The flowchart (Figure 3) shows the subsequent stages of the proposed solution for the pre-processing of the *PAN* image. The Logarithmic-Laplace filtration (LL) was used to detect the edges in the image after up-sampling and denoising (***PAN*’**). The application of logarithmic function during filtration highlighted the narrow edges that initially did not stand out on the background. Then, the conditional function based on quantiles allowed for the selection of edges of significant importance and the elimination of edges that do not bring substantial information about spatial details. The image closing process (the erosion of dilatation) not only eliminated small and irrelevant elements of detected edges but also allowed to obtain the continuity of some narrow edges. In this way, a mask with edges was developed, which formed the basis for a decision on how to modify pixels of the PAN image. If the value of the pixel did not exceed the third quartile and the value of the pixel in the original denoised image, then the pixel value of the *PAN* image did not change. When one of the conditions was not fulfilled, the new pixel value was calculated as the logarithm of the pixel value in the denoised image. The average of these images gave a modified panchromatic image, which was then used in pansharpening process. The downsampling process allowed to return to the original pixel size. 

The processing results are affected by the input settings. We used 16-fold resampling because, as we mentioned earlier, it allows preserving edge continuity than when using 4-fold resampling. While a higher sampling rate would significantly increase processing time, but would not improve edge continuity. The 4-fold resampling time for 1200 by 1200 pixels image is 0.1 s, for 16-fold resampling, it is 0.5 s, and for 32-fold resampling, it is 3.3 s. The kernel size for LL filtration was proposed according to the study [39]. The smaller the kernel size, the smaller the elements would be sharpened. Therefore, if the kernel is too small, the image with edges will only show noise and will not bring further information about the objects’ edges. On the other hand, the excessively large kernel may cause too much generalisation of edges and loss of some narrow lines and small objects. It seems that the proposed size is optimal. The next input parameter is a structural element for image closing. The larger structural element, the greater the objects’ generalisation. The minimum size of the structural element has been proposed, as the morphological closing on the image is used to eliminate erroneous elements resulting from noise. Such features are small. The larger the size of the structural element, the larger details will be removed and/or filled. The too large structural element can lead to the removal of small or narrow objects and also to the connection of objects located close to each other.

The modified panchromatic image was used for pansharpening with several methods of different specifics (PCA, GS, GIHS, HPF, WAVE, MTF_GLP and PRACS) and examined how it affects the change of spectral quality and the inetrpretative ability. Tests were performed for each data set. PCA, GS and MTF_GLP are based on component substitution, GIHS is a developmnet of Intensity-Hue-Satiration tarnsformation and this method also are based on component substitution. HPF and wavelet transformation belongs to the multiresolution group and PRACS is a hybrid method.The GS and GIHS methods were tested in three variants, including previous studies [30,32]. GS2 and GIHS2 use a double modification of the panchromatic image. The inetrpretative potential is first raised using the approach proposed in this article, and then the resulting image is modified using the approach proposed in [30], where the modified panchromatic image is the weighted average of the original *PAN* image and the intensity of the MS image. The weight is calculated for each pixel based on a comparison of the spatial information contained in that pixel. The first component of Principal Component Analysis is used for this purpose. An additional factor of 0.1 has been introduced there to ensure that sufficiently high spatial quality is maintained. This solution significantly improves the spectral quality of the data fusion and allows for the visual recognition of all objects. GS3 and GIHS3 also use a double modification of the panchromatic image. The spatial quality first increases with the approach proposed in this article, and then the resulting image is modified using the approach proposed in [32], where the goal is to improve the spectral quality of the fusion with the least possible change in spatial quality. It was proposed to use a mask that contained spatially significant objects, which were identified based on a comparison of local statistics of differences between the panchromatic image and the multispectral image intensity. The modified panchromatic image is the weighted average of the original *PAN* image and the intensity of the MS image, where the weights are a function of the pixel distance from the nearest pixel belonging to the mask with spatial details. In our research, we propose a similar approach, where the panchromatic image is modified before pansharpening, but we focus on improving object detection. We strive for pre-processing to enhance the ability to recognise objects in an image after a fusion, especially after combining custom data with a large GSD ratio. Our approach is inspired by the mentioned research but has a different purpose. The new panchromatic image is the average of its original form and its modified form, and the mask with edges is used to identify the pixels that are subject to modification. 

The other methods were chosen so that the impact of the modified image on the results of fusions with different methods could be investigated. Simple methods, which are the basis for many more advanced solutions have been chosen, so the results should be similar for more sophisticated methods based on the same principles. The quality of images was assessed based on dedicated indicators. They are based on the study of the similarity between the image after the merger and the original multispectral or high-resolution image, respectively for the spectral and spatial quality.

## 4. Results

### 4.1. Spatial Quality Assessment

**Figure 4:**
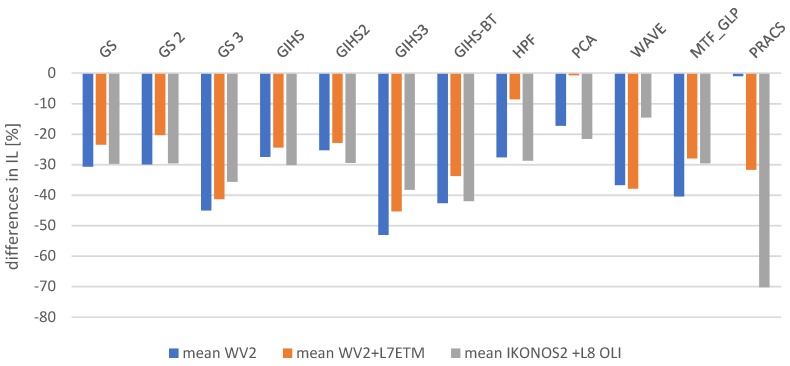
The difference in IL after comparing the values from the classical and modified approach expressed as a percentage in relation to the values from the classical approach.

Spatial quality was assessed using the Laplacian Index IL% [41] The assessment was made for all spectral channels in the visible, near-infrared and mid-infrared range for each tested dataset. The graphs (Figure 4) show the average change in the IL% for the tested datasets. These values determine the change that occurred after applying the panchromatic image modification (mod) used in the pansharpening process in relation to the classical approach (class).

**Figure 5:**
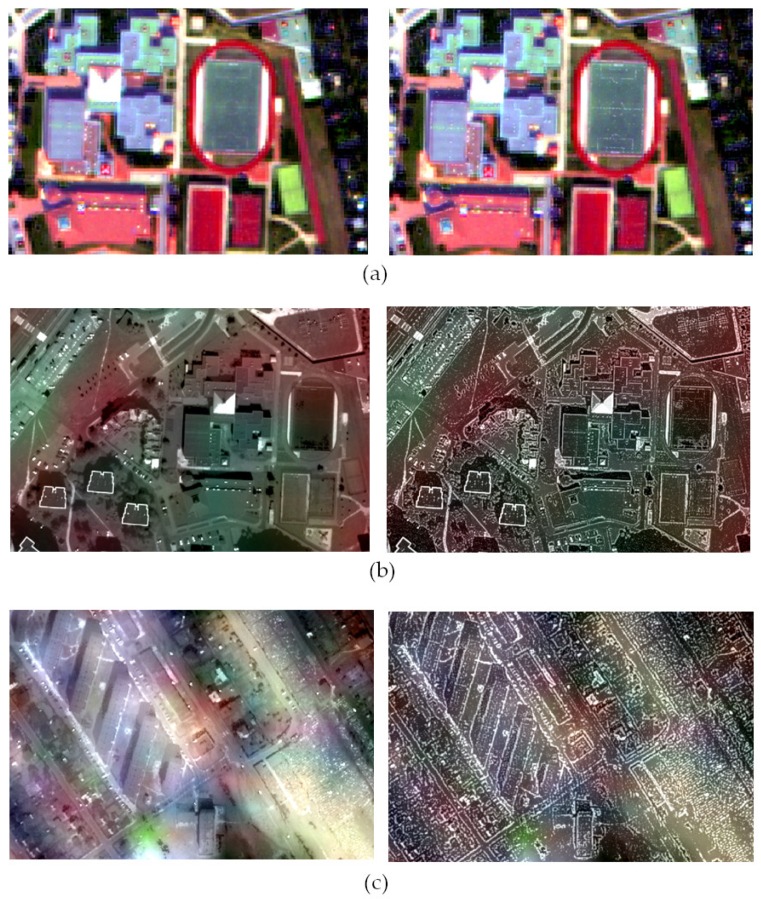
Results of pansharpening using the GIHS3 method in the classic approach (left) and modified (right) images acquired by satellites (**a**) WorldView-2 (**b**) WorldView-2 and Landsat 7 ETM (**c**) IKONOS-2 and Landsat 8 OLI.

**Figure 6:**
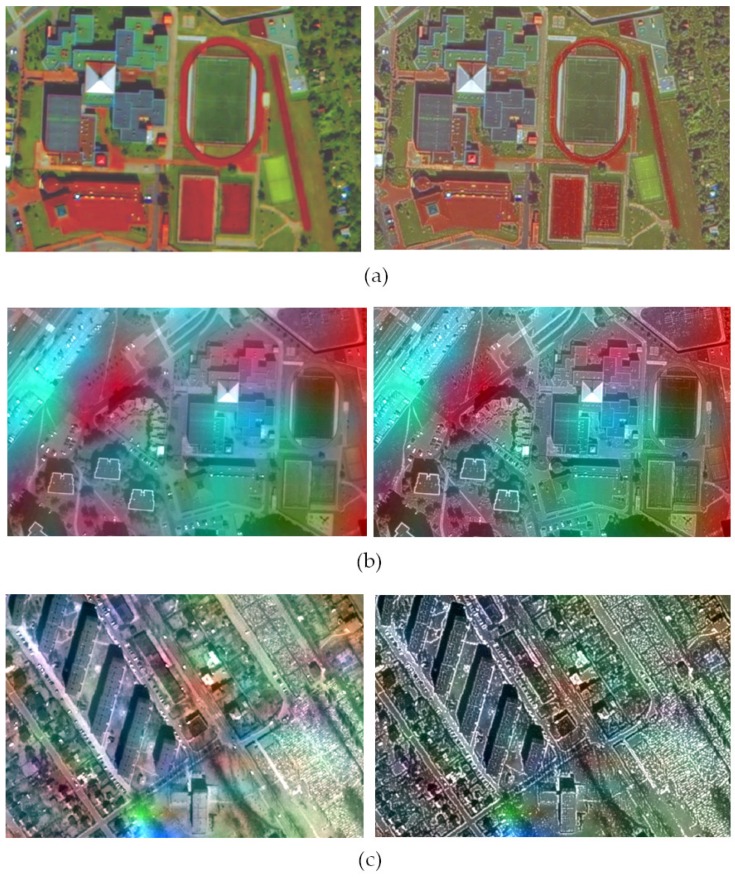
Results of pansharpening using the PCA method in the classic approach (left) and modified (right) images acquired by satellites (**a**) WorldView-2 (**b**) WorldView-2 and Landsat 7 ETM (**c**) IKONOS-2 and Landsat 8 OLI.

IL% index values in subsequent spectral bands decreased after applying a modified panchromatic image, which suggests deterioration of the interpretative potential. Figure 5 shows that spatial quality has deteriorated by up to 70% in some cases, which should be seen as a fuzzy image. However, the visual analysis itself shows that the observer can identify with the naked eye more details in the image (Figure 5 and Figure 6). Such discrepancy results from the method of calculating the used metric. Processing the *PAN* image to sharpen the edges caused brightness changes in pixels, which in turn resulted in a decrease in the correlation between the sharpened image and the original panchromatic image. For this reason, classic indicators for assessing spatial quality do not reflect the interpretative ability of the image. That is why we proposed an indicator based on the SURF detector (Section 4.2).

### 4.2. Features-Detector-Based Quality Index

Most of the methods for assessing the spatial quality of the image are based on the correlation coefficient (CC) or Root Mean Square Error (RMSE) between the compared images sharpened with a high-pass filter, which in fact comes down to testing the similarity of the corresponding pixels. This approach excludes the possibility of improving the spatial qualities of the image. Every modification of a high-resolution image is perceived as a deterioration of quality, which is not always true. Sharpening details in image results in the possibility of recognizing more features (which is important mainly for automatic algorithms), but also changes the brightness of the corresponding pixels. That, in turn, causes a decrease in similarity and deterioration in the value of indicators. That is why we propose a new index for pansharpening quality assessment (Features-detector-based Quality Index $Q_{FD}$), which not only takes into account pixel brightness but also image geometry and topological relationships. The $Q_{FD}$ indicator uses image detectors that are based on finding characteristics (pixel configurations) such as lines, edges, corners, the line ends, textures, clusters, ridges, and skeletons. The proposed indicator does not assess the similarity between the shot and the original image based on pixel values, but allows you to determine the image value in object detection.

One of the more straightforward and efficient algorithms that detect characteristics is SURF (Speeded Up Robust Features) [42]. Its detector is based on Hessian (Equation (4)), while the descriptor is based on the position and placement of points in the image:

(4) $$H\left(x,y\right)=\left[\begin{array}{cc} L_{xx}\left(x,\sigma \right) & L_{xy}\left(x,\sigma \right)\\ L_{yx}\left(x,\sigma \right) & L_{yy}\left(x,\sigma \right) \end{array}\right]$$

where L is a convolution of the second derivative of the Gaussian function on a given scale σ with the input image. In the implementation of the second-order Gaussian filters are replaced by an approximation using box filter, which can be extremely efficiently using the so-called integral image II (Equation (5)) [43]:

(5) $$II\left(x,y\right)=\;\overset{x}{\underset{i=0}{\sum }}\overset{y}{\underset{j=0}{\sum }}I\left(x,y\right)$$

The evaluation of filters guarantees scalability at different scales, which, together with the local maxima, creates a set of detected features. Coefficients of Haar filter are used to detect the dominant image orientation of each feature, which guarantees the rotation of the invariance. Position, scale and orientation are characterised by a descriptor calculated for each characteristic [43]. The characteristic points detected by the SURF detector correspond to the spatial details in the image. A larger number of points can suggest greater possibilities in the interpretation and detection of objects in the image. The index $Q_{FD}$ is the quotient of the number of characteristic points $N_{k}$ in the channel k after the fusion to the number of characteristic points on the high-resolution panchromatic image $N_{PAN}$:

(6) $$Q_{FD}=\;\frac{N_{k}}{N_{PAN}}$$

The ratio can theoretically take values in the range (0, ∞). The larger the value, the more characteristic points were detected in the image after the fusion concerning the number of points in the *PAN* image. Practically, the index value does not take values greater than 1, but the upper range limit can not be set. Depending on the techniques used, there is always a chance that in the sharpened image, it will be possible to identify more details than in the original panchromatic image. 

This effect is possible mainly after applying highpass filtration and similar methods that extract the edges from the background, allowing, for example, better identification of object corners. Image detectors are very sensitive to radiometric quality. In order to reliably compare the number of points identified in the *PAN* image and post-fused MS image, it is recommended to adjust the radiometry of the images by equalization of the *PAN* image histogram and matching the histograms of the subsequent image channels after the fusion.

**Figure 7:**
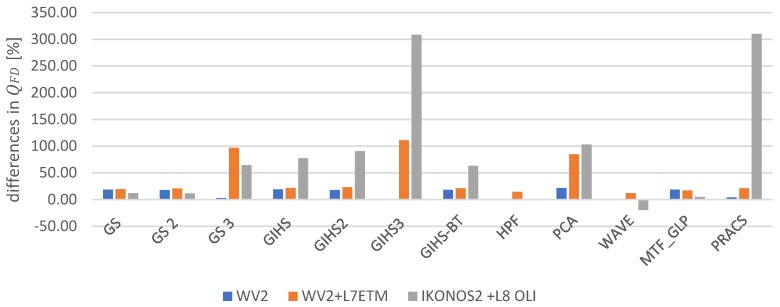
The difference $Q_{FD}$ after comparing the values from the classic and modified approaches expressed as a percentage in relation to the values from the classical approach.

The interpretative potential was assessed using the proposed $Q_{FD}$ indicator. The assessment was made for all spectral channels in the visible, near-infrared and mid-infrared range for each tested dataset. The graphs (Figure 7) show the average change in the $Q_{FD}$ for the tested datasets. These values determine the change that occurred after applying the panchromatic image modification (mod) used in the pansharpening process in relation to the classical approach (class).

The highest increase was observed for GS3, GIHS3 and PCA methods and for PRACS method for IKONOS. Table 1, Table 2 and Table 3 summarise the values of the $Q_{FD}$ index for channels in the visible, near-infrared and mid-infrared range of each dataset. For data from WorldView-2, the biggest improvement was observed after applying the PCA method (21.4% in relation to the values from the classical approach). A similar improvement (on average by 18%) was for the majority of methods except for GS3 and GIHS3, which used a panchromatic image modified earlier in order to preserve the spatial qualities of the image [32]. The GS, GS2 and PCA methods achieved very high values of indicator. The index $Q_{FD}$ is greater than or equal to 1, which means that in the modified approach, more characteristic points were detected than in the original panchromatic image. In many cases, values have risen above 0.80, which indicates a high level of spatial information recorded in the image after the merger.

After the integration of data from set 2, the values of the index are lower (Table 2), which is caused by high spatial resolution ratio. When data is integrated with a GSD ratio of 1:60, the resulting images are blurred, which makes it challenging to detect characteristic points. The level of blur depends, of course on the fusion method, in some cases, the image is sharp enough, and the interpretative potential is relatively high, in other cases high spectral quality is guaranteed. Then the colours are correctly mapped, but the image is not sharp. In the case of set 2, very good results were obtained when the modified approach of the GS3 method and all methods based on GIHS was applied. The GIHS methods gave good spatial results already in the classical approach, and the modified image application still improved these results. The highest improvement (over 100% in relation to the values from the classic approach) took place for PCA, GS3 and GIHS3 methods. Whereas the PCA method in the classical approach gave a relatively low quality, which improved significantly after applying the modified image (by 177% in the first channel, 77 to 94% in channels 2–4, and by 23% and 46% in channels 5 and 6).

Similarly, for set 3 (Table 3), the highest increase of the $Q_{FD}$ index occurred for methods based on GIHS (especially GIHS3) and PRACS. In this case, also the modification of methods GS3, GIHS3, PCA allowed detecting more characteristic points than in the panchromatic image. In the output image after the GIHS3 and PRACS fusion, the $Q_{FD}$ index increased by over 300%. That is a very high value, and it results from the fact that during the integration of data from the IKONOS-2 and Landsat 8 OLI satellites, the GIHS and PRACS methods did not give good results, and their modifications decreased the spatial quality. However, modifying the *PAN* image following the proposed approach allows for a significant improvement of these results.

The table does not show results for the WAVE method because with the pixel size of the *PAN* 1 m image and the large GSD ratio, the wavelet transform method gave a very fuzzy image with a strong jagging effect, and the spatial elements were difficult to identify. Therefore, very few characteristic points were detected and it was not possible to reliably evaluate changes after applying both approaches. The values of $Q_{FD}$ index are consistent with the visual analysis.

The results of the visual analysis are covered by the results of the $Q_{FD}\;$ indicator (Figure 7). In most cases, the value of the index increased, which means that it was possible to identify a greater number of characteristic points. Figure 5 and Figure 6 shows the results of pansharpening using PCA and GIHS3, where the index $Q_{FD}$ changed most respectively for sets 1, 2 and 3. The sharpness of the image has significantly improved, especially this effect is noticeable when fusing data with a high GSD ratio. It is possible to identify more land cover details. In the original parts of the scenes from the World View-2 satellite, the line on the pitch can not be seen, but after applying the modified *PAN* picture, they are clearly visible. Similarly, it is easier to identify the outline of buildings, road edges, narrow paths and other objects.

**Figure 8:**
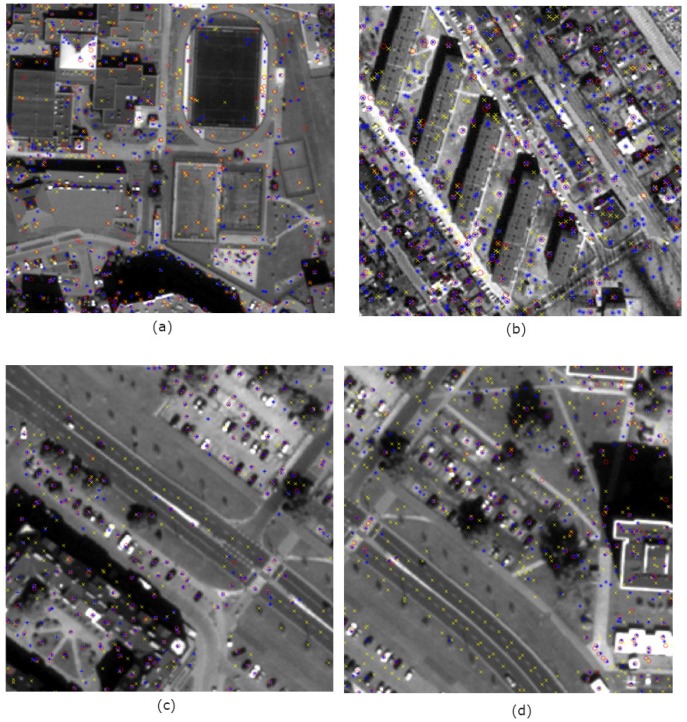
Characteristic points detected in: the original panchromatic image (marked with a blue star), the image after the fusion according to the classic approach (red circle) and in the image after the image fusion according to the modified approach (yellow cross) (**a**) for fusion WorldView-2 *PAN* and WorldView-2 MS (PCA) (**b**) for fusion IKONOS 2 *PAN* and Landsat 8 OLI (GIHS 3) (**c**) for fusion WorldView-2 *PAN* and Landsat 7 ETM (PRACS) (**d**) for fusion WorldView-2 *PAN* and Landsat 7 ETM (GS 3).

Figure 8 presents characteristic points detected in the original panchromatic image (marked with a blue star), characteristic points identified in the image after the fusion according to the classic approach (red circle) and characteristic points detected in the image after the image fusion according to the modified method (yellow cross). Many points are located in similar positions on each image, but there are also differences in the number and location of points detected in subsequent images of the same area. Characteristic points identified in the original *PAN* images occur in different places; they do not always represent essential details. In the fused images, points were found in other locations than on the *PAN* image, which is the result of different radiometry of the images, and therefore features determination was affected. In the image after the fusion in the classical approach fewer points representing easily identifiable details were detected than in the *PAN* image. However, after a fusion using the modified approach, the number of characteristic points generally increases and sometimes exceeds the number of points in the *PAN* image.

An increase in $Q_{FD}$ values over one may suggest the occurrence of erroneous lines resulting from the use of image sharpening techniques. The presence of such errors cannot be completely eliminated; however, it is clear from Figure 8 that an index value higher than one does not necessarily mean a significant number of artificially sharpened edges, and translates into a greater number of characteristic points detected in the images. Only after applying the modified approach, characteristic points of pitch lines, road lines, narrow paths, chimneys on buildings and others were detected. That proves that there are greater possibilities for automatic detection of objects. Therefore, the $Q_{FD}$ index higher than 1 occurs when pre-processing methods allow for a local improvement of image radiometry in places where spatial details appeared in the original *PAN* image that did not stand out sufficiently from the background.

### 4.3. Image Segmentation

The results of image segmentation also confirm the improvement of spatial quality in terms of object detection after fusion. Image segmentation is often the preliminary stage of automatic object detection and Object-Based Image Analysis (OBIA). Therefore, the results of segmentation bring information about the functional properties of the proposed solution.

**Figure 9:**
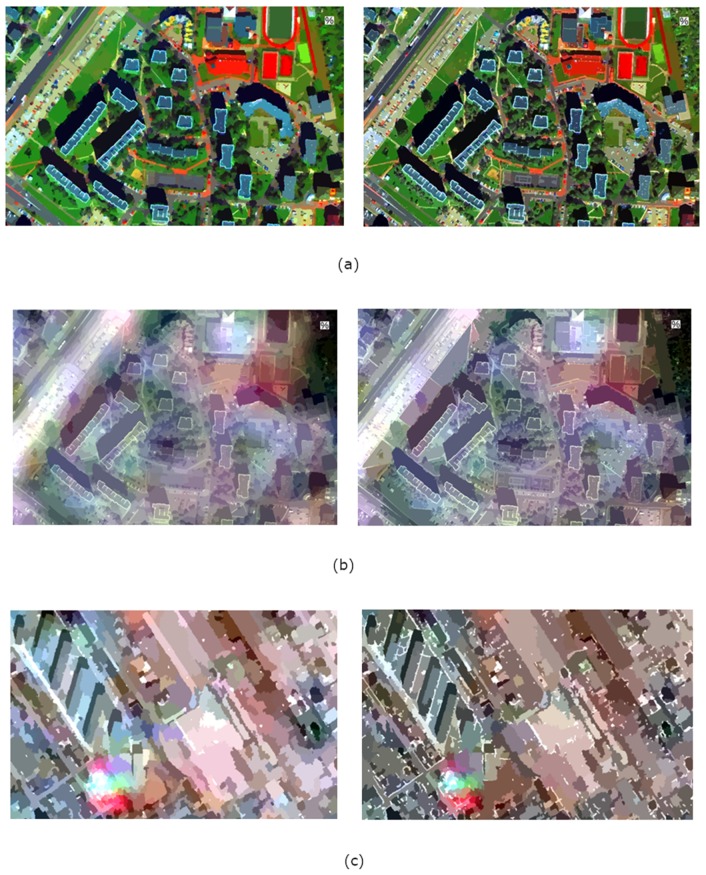
Results of image segmentation after pansharpening using the PCA method in the classic approach (left) and modified (right) for images acquired by satellites (**a**) WorldView-2 (**b**) WorldView-2 and Landsat 7 ETM (**c**) IKONOS-2 and Landsat 8 OLI.

Figure 9 shows the results of segmentation (in natural colours) performed on images after fusion using the PCA method in a classic and modified approach. The solutions used in the modification of the panchromatic image allow for better identification of the objects’ edges, which is the basis for object discrimination during the segmentation process. The higher the spatial resolution of the panchromatic image, the greater the potential for segmentation improvement. The visibility of the edges in the high-resolution image allowed for easy detection of road lines, details on the buildings’ roofs and distinction of the pitch parts (Figure 9a,b). In addition, edges sharpening allows for better identification of object shapes, which can be mainly observed for buildings (Figure 8c). The shapes are more regular and match the real ones. The modification of pixel of the edges allowed the automatic detection of some narrow paths between buildings, which after fusion in the classical approach were challenging to identify even for the skilled analyst.

Synthetic ground truths [44] were used to quantify the segmentation results. In each image, several dozen random points belonging to different classes of the ground truth were selected, and their membership to classes after the segmentation was compared. The following classes were considered: buildings, pavements and paths, roads, road lines, lawn, trees, parking lots and playgrounds. Table 4 summarises the correlation coefficients describing the correspondence between ground truth and the result of segmentation for an exemplary set of images after fusion by the PCA method. 

The pre-processing of the panchromatic band had a positive effect on increasing the quality of segmentation. Segments have more regular shapes, which are more similar to the ground truth. There is also a clear relationship between the GSD of high-resolution image and processing results. Both when testing the number of characteristic points and when testing the quality of segmentation, a much more significant improvement can be seen for data sets using the image with a higher spatial resolution (0.5 m). In the case of images from the IKONOS satellite, the object detection capabilities have also improved, but there are even more misidentified objects. On the results of the fusion of the multispectral image with the WorldView-2 panchromatic image, the possibility of narrow linear objects detection has definitely improved. Unfortunately, in the case of set 3, the results are slightly worse. First of all, the lower spatial resolution made it impossible to recognise some objects, such as road lines. Secondly, the ability to recognize narrow pavements near blocks of flats has improved, but similar lines that occur in single-family housing were detected as erroneous edges, which were the result of high-pass filtration. It is a result that the proposed approach is mainly dedicated to the integration of low-spectral multispectral data with very high-resolution panchromatic data (pixel size below 1 m).

### 4.4. Spectral Quality Assessment

**Figure 10:**
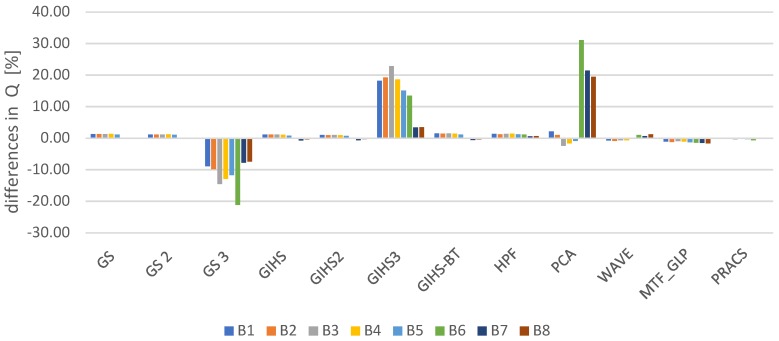
The difference in the value of the Q ratio in subsequent image bands (B1, 2, …, 8) for different pansharpening methods after comparing the classical approach and modified for the data obtained by WorldView-2 (*PAN* and MS) expressed as a percentage of the values from the classical approach.

**Figure 11:**
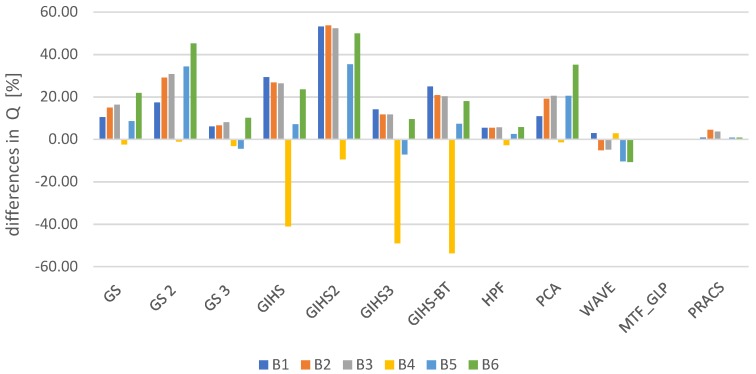
The difference in the value of the Q ratio in subsequent image bands (B1, 2, …, 6) for different pansharpening methods after comparing the classical approach and modified for the data obtained by WorldView-2 (*PAN*) and Landsat 7 ETM (MS) expressed as a percentage of the values from the classical approach.

**Figure 12:**
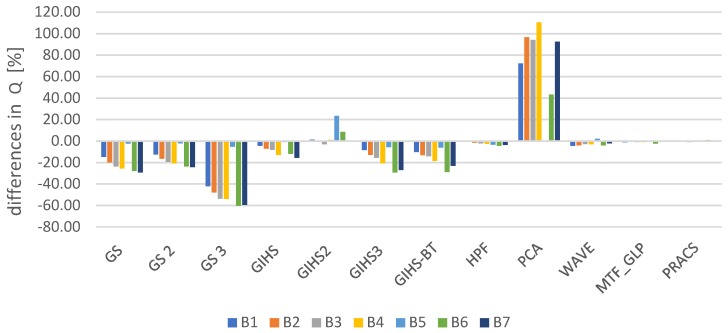
The difference in the value of the Q ratio in subsequent image bands (B1, 2, …, 7) for different pansharpening methods after comparing the classical approach and modified for the data obtained by IKONOS-2 (*PAN*) and Landsat 8 OLI (MS) expressed as a percentage of the values from the classical approach.

**Figure 13:**
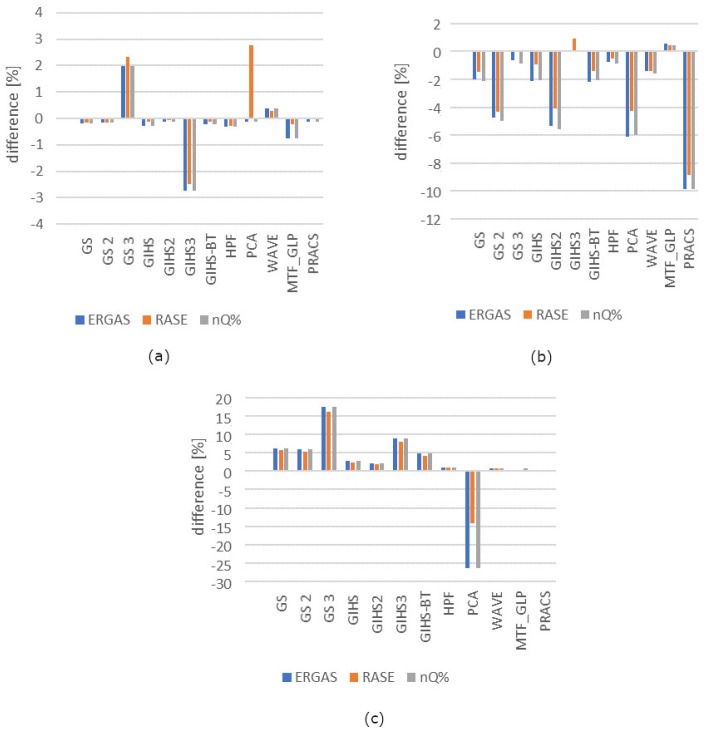
The difference in the value of the ERGAS, RASE and nQ% for different pansharpening methods after comparing the classical approach and modified for the data obtained by IKONOS-2 (*PAN*) and Landsat 8 OLI (MS) expressed as a percentage of the values from the classical approach for datasets (**a**) WorldView-2 (**b**) WorldView-2 and Landsat 7 ETM (**c**) IKONOS-2 and Landsat 8 OLI.

In this article, we focused mainly on improving the interpretative potential but the spectral quality cannot be omitted entirely. This is a very important aspect because the high spectral quality allows for precise reproduction of reflective properties of objects in different spectral ranges. For this purpose, the Universal Image Quality Index (Q) indicator [45]-based on the correlation coefficient and Relative Dimensionless Global Error (ERGAS) [46], Relative Average Spectral Error (RASE) [47] and newQuality (nQ%) [48]-based on RMSE value. The metrics were counted for each image band after fusion in each data set (Figure 10, Figure 11, Figure 12 and Figure 13). Previously, images were prepared for evaluation according to the Wald protocol [46,49,50]. Usually, the improvement of spectral quality results in deterioration of spatial quality and vice versa [51]. In order to precisely reproduce the spatial details, it is necessary to maintain the brightness of the pixels guaranteeing high contrast in the area the edges of the objects. Such pixel values deviate from their response to multispectral imaging, which results in lowering the overall spectral quality of the image in subsequent channels.

The results of data integration from set 3 have kept the tendency consistent with the abovementioned relationship. The improvement of spatial quality caused a decrease in spectral quality regardless of the method used. In set 2, this was the case only for the GIHS method. In other cases, spectral quality has improved. Similarly, in set 1, in the majority of cases, the increase of the value of the Q indicator and the decrease of the ERAGS, RASE and nQ% are visible. For data with a standard GSD ratio, the changes were not very large (except for the GIHS3 method). In extreme cases, the changes in the Q ratio increased by about 20% compared to the classical approach and the changes in the others indicators decreased by about 2% compared to the classical approach. The integration of data with a high spatial resolution ratio is more sensitive to changes depending on the fusion methodology and results in more significant quality changes. In the case of set 2, the improvement of spectral quality reached even up to 50% for the GIHS2 method, and on average, it was about 10% of the value from the classical approach. In turn, indicators based on RMSE showed improvement to about 5% for GS2, GIH2 and PCA and about 10% for PRACS.

The different results for the three sets result from the different specificity of the terrain and the various histograms of the original panchromatic images. The *PAN* image acquired from the satellite IKONOS was characterised by a low standard deviation (it was 8.1), while the standard deviation of the *PAN* image was 20.8. The higher the variety of pixel values in the image, the less harmful to the interpretative potential will be the modification of the *PAN* image. Due to the low contrast of the *PAN* image from the IKONOS satellite, sharpening of the spatial objects was very noticeable, which resulted in significant changes in the image statistics and decreased Q values and increased of others indicators. For images with greater variance, better results were observed-both spatial and spectral quality improved, which is a positive effect in the context of imaging of urban areas.

### 4.5. Accuracy Assessment

After the image fusion the accuracy was assested. For this purpose, the coordinates of a set of evenly distributed points were measured on each pansharpened image (both in classic and modified approaches). Before processing, the images were subjected to initial geometric correction, so the coordinates on the images were measured in the UTM system (North, 34 Zone). The measured coordinates were compared with the actual values and based on this the Total RMSE was calculated for each image (Table 5).

The absolute values of Total RMSE in both approaches usually differed from about 0.1 m to 0.2 m for the integration with the WorldView-2 *PAN* image and from 0.1 m to 0.6 m for the combination with the IKONOS-2 *PAN* image. These values reach up to half the pixel size of the high-resolution image, so these changes can be considered insignificant and do not change the spatial accuracy of the image. Only for the case when the wavelet transformation (WAVE) was applied, the proposed solution generally resulted in an increase of accuracy to about 1 pixel. The proposed approach did not use image processing methods that changed image geometry. Only the application of up-sampling and down-sampling could result in small image shift due to the interpolation. Changes in accuracy may vary slightly depending on the resampling methods used. The proposed solution for pre-processing of a panchromatic image does not significantly change the accuracy of the image, but allows easier identification of objects. The image is not only more readable and clear for visual analysis but also allows for more precise detection of objects when semi-automatic methods are used, e.g., segmentation.

## 5. Discussion

So far, some methods have already included enhancement of high resolution image details, but this was done either by taking more information from the panchromatic image during fusion or by high-pass filtration built into the fusion algorithm (dedicated to one selected method) [52]. This manuscript proposes a universal solution, adequate for most methods, where the panchromatic image is modified in the stage preceding the pansharpening with any method.

**Figure 14:**
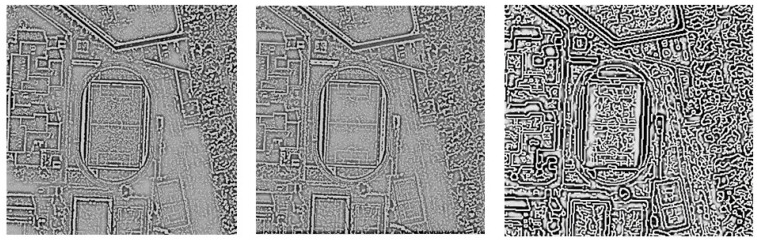
From the left, proposed filter, Laplace filter, Canny filter.

The authors proposed a new approach to image filtration for edge detection. This approach allows detection of all edges, regardless of the direction without modifying their shape (unlike the Canny filter). The Canny filter strongly exposes all detected edges and also has a directional effect. The use of gradients operates in a directional way and does not allow the detection of narrow edges in any directions. Non-directional edge detection uses Laplace filter, but the Laplace filter itself cannot detect narrow edges, which is possible after applying the approach proposed in this article (Figure 14).

The proposed method of filtration is based on the Laplace filter; therefore it detects the edges very similarly. However, its continuity is more strongly preserved and, additionally, the use of logarithms increases the local contrast near the edges, which makes them more visible and easier to detect. The main goal of the research was to improve the interpretative potential of pansharpening results, mainly when combining data with a high GSD ratio. Typically, correlation-based or RMSE-based metrics are used to assess spatial quality, which compare the original high-resolution image with the image after the fusion, after prior high-pass filtration. However, these methods are not always reliable, especially if the panchromatic picture undergoes certain modifications during the integration process. Therefore, a new indicator was proposed, which is based on image detectors and examines not only the brightness of pixels but also the geometry and topology in the image. This approach allows comparing the number of characteristic points detected in the images before and after the merger. The research has shown that the proposed method of *PAN* image modification increases the interpretative potential of images after the fusion and works more strongly for data with a high GSD ratio. In the case of combining data from the same sensor with the standard ratio of the spatial resolution, the interpretative potential improved by a dozen or so per cent, while for data with a high GSD ratio, the improvement was several dozen or even several hundred per cents. Such large changes are proof of a significant improvement in images, which in the classic approach were strongly blurred. It is worth noting that for the PCA method and submission of modifications from [30,32] and modifications from this study for GS and GIHS methods in sharpened images can detect more points than in the original panchromatic image. It is worth noting that for high-resolution data from the WordView-2 satellite, the proposed approach raised not only the interpretative potential also spectral quality, and in some cases where a single modification from previous research [30,32] did not give good results, combining it with the method proposed in this article significantly improved the results. The improvement of both types of quality is a very good effect, so far various studies have attempted to achieve a compromise between spectral and spatial quality [22,53,54]. 

The proposed approach can significantly facilitate the automatic vectorisation of selected objects, segmentation, object classification or even fitting a series of images to each other based on characteristic and homologous points. In addition, the submission of two modifications is an excellent way to improve the quality of data fusion with a high GSD ratio both in terms of spectral and spatial quality without compromising image accuracy. In presented studies, a new approach to pre-processing the panchromatic image before fusion with a low-resolution multispectral image was proposed, without considering the Uncertainty-Aware Visual System. However, according to research by Gillmann et al. [55], each image voxel is subject to uncertainty. During pre-processing, these uncertainties may vary depending on the method used or the sequence of methods used. Consideration of uncertainty can lead to improvement of the quality of the output image, and further assist in the automatic detection of objects. Moreover, including different weights during Laplace filtration, depending on the uncertainty of each pixel, would probably help eliminate minor misalignment. However, as the authors of the research themselves indicate, the uncertainty term is not fully defined [55]. Therefore further research is needed to develop this issue.

In further research, it is worth considering the aspect mentioned above of image uncertainty. Moreover, in further research it is planned to use neural networks for local modification of the panchromatic image and correction of its radiometric quality. It should be borne in mind that the better the radiometric quality of the input images, the better the results, so contrast modulation or radiometric correction can be applied during data preparation.

## 6. Conclusions

This manuscript proposes a new approach to the fusion of differential resolution images, which aims primarily at improving the interpretative potential A modification of the panchromatic image based on Laplace filtration and logarithmic function has been proposed. In addition, a new indicator for interpretative potential assessment based on the SURF feature detector was proposed, which examines not only the brightness of pixels but also the geometry and topology in the image. That allows for the comparison of the number of characteristic points detected in the images before and after the data fusion. The proposed approach was examined for three sets of data with different GSD ratios, one of which had a standard ratio of 1:4, and the other sets combined data with a significant difference in resolution. Each set was examined using several different pansharpening methods, taking into account also the modifications proposed in other publications. The results showed that the proposed approach significantly increases the interpretative potential of post-merger images, especially for data with a high GSD ratio. While combining data from the same sensor (with a standard ratio of the spatial resolution) the interpretative potential improved by several per cents. For data with a high GSD ratio, the improvement was several dozen, or even several hundred per cents for blurred images after fusion (if the result of the fusion in the classic approach were highly blurred images). The proposed approach can be the starting point to develop other methods of panchromatic image modification; their composition may lead to an increase in both spectral and spatial quality, which in turn may facilitate the automatic vectorisation, classification or even the mutual matching of a series of images.

## Figures and Tables

**Table 1:** $Q_{FD}$ index values for data acquired from WorldView-2 (*PAN* and MS).

Method	b1	b2	b3	b4	b5	b6	b7	b8
GS class	0.75	0.80	0.93	0.88	0.86	0.92	0.70	0.67
GS mod	**0.94**	**1.00**	**1.16**	**1.10**	**1.07**	**1.03**	**0.74**	**0.71**
GS 2 class	0.73	0.77	0.89	0.85	0.84	0.89	0.68	0.65
GS 2 mod	**0.91**	**0.96**	**1.10**	**1.06**	**1.03**	**0.99**	**0.72**	**0.69**
GS 3 class	0.72	0.76	0.87	0.83	0.83	0.84	0.66	0.63
GS 3 mod	**0.75**	**0.80**	**0.90**	**0.87**	**0.86**	0.84	0.66	0.62
GIHS class	0.56	0.58	0.62	0.62	0.59	0.88	0.65	0.64
GIHS mod	**0.71**	**0.71**	**0.80**	**0.74**	**0.70**	**1.01**	**0.72**	**0.70**
GIHS 2 class	0.55	0.57	0.60	0.60	0.59	0.86	0.63	0.63
GIHS 2 mod	**0.68**	**0.69**	**0.76**	**0.72**	**0.68**	**0.96**	**0.70**	**0.69**
GIHS 3 class	0.61	0.62	0.66	0.67	0.64	0.85	0.63	0.63
GIHS 3 mod	0.61	0.62	0.66	0.65	0.63	0.84	0.61	0.62
GIHS BT class	0.54	0.55	0.62	0.63	0.60	0.93	0.70	0.73
GIHS BT mod	**0.66**	**0.66**	**0.76**	**0.74**	**0.71**	**1.04**	**0.81**	**0.83**
HPF class	0.89	0.90	0.99	0.98	0.93	1.05	0.95	0.94
HPF mod	**0.91**	**0.91**	**1.00**	**0.98**	**0.95**	**1.06**	0.94	0.93
PCA class	0.69	0.70	0.92	0.94	0.78	0.67	0.55	0.55
PCA mod	**0.97**	**1.00**	**1.11**	**1.18**	**1.07**	**0.70**	0.55	0.55
Wave class	0.89	0.96	0.96	0.92	0.93	1.17	0.92	0.90
Wave mod	**0.90**	**0.97**	0.96	0.92	0.93	**1.19**	0.92	0.89
MTF_GLP or	0.69	0.70	0.77	0.76	0.76	0.91	0.80	0.83
MTF_GLP mod	**0.85**	**0.86**	**0.94**	**0.87**	**0.92**	**1.03**	**0.93**	**0.97**
PRACS or	0.55	0.56	0.60	0.60	0.58	0.74	0.60	0.60
PRACS mod	**0.57**	**0.58**	**0.66**	**0.55**	**0.63**	**0.79**	**0.62**	**0.63**

**Table 2:** $Q_{FD}$ index values for data acquired from WorldView-2 (*PAN*) and Landsat 7 ETM (MS).

Method	b1	b2	b3	b4	b5	b6
GS class	0.64	0.82	0.77	0.61	0.92	0.90
GS mod	**0.76**	**0.98**	**0.92**	**0.71**	**1.11**	**1.07**
GS 2 class	0.55	0.69	0.65	0.54	0.78	0.76
GS 2 mod	**0.66**	**0.85**	**0.79**	**0.63**	**0.95**	**0.91**
GS 3 class	0.44	0.57	0.54	0.44	0.69	0.65
GS 3 mod	**0.85**	**1.18**	**1.11**	**0.72**	**1.40**	**1.34**
GIHS class	0.84	0.83	0.80	0.78	0.80	0.82
GIHS mod	**1.00**	**1.00**	**0.97**	**0.96**	**0.98**	**0.99**
GIHS 2 class	0.80	0.80	0.76	0.74	0.75	0.78
GIHS 2 mod	**0.99**	**0.98**	**0.94**	**0.91**	**0.93**	**0.95**
GIHS 3 class	0.71	0.71	0.67	0.63	0.66	0.68
GIHS 3 mod	**1.45**	**1.46**	**1.42**	**1.36**	**1.42**	**1.43**
GIHS BT class	0.84	0.84	0.79	0.82	01	0.80
GIHS BT mod	**1.00**	**1.00**	**0.97**	**0.98**	**0.98**	**0.98**
HPF class	0.24	0.23	0.24	0.23	0.23	0.23
HPF mod	**0.28**	**0.27**	**0.27**	**0.27**	**0.26**	**0.26**
PCA class	0.07	0.26	0.21	0.26	0.90	0.51
PCA mod	**0.21**	**0.46**	**0.41**	**0.49**	**1.11**	**0.74**
Wave class	0.46	0.48	0.47	0.44	0.46	0.51
Wave mod	**0.53**	**0.53**	**0.54**	**0.50**	**0.49**	**0.55**
MTF_GLP or	0.67	0.67	0.66	0.67	0.65	0.65
MTF_GLP mod	**0.92**	**0.92**	**0.92**	**0.05**	**0.90**	**0.91**
PRACS or	0.78	0.83	0.78	0.16	0.61	0.51
PRACS mod	**1.15**	**1.13**	**1.09**	**0.16**	**0.91**	**0.77**

**Table 3:** $Q_{FD}$ index values for data acquired from IKONOS-2 (*PAN*) and Landsat 8 OLI (MS).

Method	b1	b2	b3	b4	b5	b6	b7
GS class	0.77	0.79	0.87	0.81	0.03	0.76	0.85
GS mod	**0.85**	**0.88**	**0.98**	**0.92**	**0.02**	**0.85**	**0.96**
GS 2 class	0.72	0.74	0.83	0.77	0.02	0.73	0.81
GS 2 mod	**0.79**	**0.81**	**0.92**	**0.86**	0.02	**0.82**	**0.92**
GS 3 class	0.55	0.56	0.63	0.58	0.03	0.53	0.61
GS 3 mod	**0.89**	**0.93**	**1.05**	**0.95**	0.02	**0.87**	**1.00**
GIHS class	0.43	0.45	0.48	0.38	0.04	0.46	0.44
GIHS mod	**0.76**	**0.79**	**0.80**	**0.72**	**0.20**	**0.81**	**0.78**
GIHS 2 class	0.35	0.37	0.38	0.29	0.03	0.38	0.34
GIHS 2 mod	**0.67**	**0.70**	**0.67**	**0.60**	**0.17**	**0.74**	**0.63**
GIHS 3 class	0.26	0.26	0.24	0.20	0.03	0.26	0.22
GIHS 3 mod	**0.98**	**1.00**	**1.00**	**0.92**	**0.37**	**1.01**	**0.97**
GIHS BT class	0.42	0.44	0.50	0.52	0.51	78	0.60
GIHS BT mod	**0.72**	**0.75**	**0.80**	**0.80**	**0.73**	**0.99**	**0.86**
HPF class	0.29	0.29	0.29	0.29	0.27	0.28	0.28
HPF mod	0.28	0.29	0.29	0.29	0.27	0.27	0.27
PCA class	0.76	0.76	0.69	0.89	0.01	0.01	0.40
PCA mod	**1.04**	**1.05**	**1.00**	**1.09**	**0.05**	**0.06**	**0.82**
MTF_GLP or	0.71	0.73	0.71	0.70	0.75	0.71	0.72
MTF_GLP mod	**0.74**	**0.75**	**0.74**	**0.36**	**0.80**	**0.76**	**0.76**
PRACS or	0.01	0.01	0.02	0.01	0.01	0.02	0.01
PRACS mod	**0.05**	**0.07**	**0.10**	0.01	0.01	**0.03**	**0.05**

**Table 4:** Correlation coefficients describing the correspondence between ground truth and the result of segmentation after fusion by the PCA method.

	WorldView-2 (PAN and MS)		WorldView-2 (PAN) and Landsat 7 ETM (MS)		IKONOS-2 (PAN) and Landsat 8 OLI (MS)	
	Class	Mod	Class	Mod	Class	Mod
Buildings	0.92	0.94	0.68	0.78	0.48	0.63
Walkways and paths	0.66	0.84	0.58	0.74	0.15	0.46
Roads	0.97	0.97	0.74	0.78	0.68	0.56
Lanes on the roads	0.20	0.86	0.20	0.88	0.00	0.00
Grass	0.98	0.97	0.90	0.92	0.66	0.64
Trees	0.90	0.91	0.71	0.75	0.34	0.37
Parking lots and playgrounds	0.88	0.92	0.77	0.86	0.36	0.39
All	0.79	0.92	0.65	0.82	0.38	0.44

**Table 5:** Differences between Total RMSE of pansharpened images (classic and modified approach).

Method	WorldView-2 (*PAN* and MS)		WorldView-2 (*PAN*) and Landsat 7 ETM (MS)		IKONOS-2 (*PAN*) and Landsat 8 OLI (MS)	
	X [m]	Y [m]	X [m]	Y [m]	X [m]	Y [m]
GS	0.1	−0.1	0.1	0.1	−0.1	0.1
GS 2	0.1	−0.1	0.1	−0.1	0.1	−0.1
GS 3	0.1	−0.2	0.1	−0.1	0.1	0.1
GIHS	0.1	−0.2	0.1	−0.2	0.1	−0.6
GIHS2	0.1	−0.1	0.1	−0.1	−0.2	−0.3
GIHS3	0.1	−0.1	0.1	0.1	−0.2	−0.1
GIHS-BT	0.2	−0.2	0.1	−0.1	0.1	0.1
HPF	0.1	−0.2	0.1	0.1	0.1	−0.4
PCA	−0.1	−0.2	0.1	−0.2	0.1	−0.3
WAVE	0.5	−0.2	0.3	0.4	0.9	1.2
MTF_GLP	0.1	−0.1	0.2	0.2	0.1	−0.1
PRACS	0.1	−0.2	0.1	−0.1	0.1	0.1
